# Association between -308G/A TNFA Polymorphism and Susceptibility to Type 2 Diabetes Mellitus: A Systematic Review

**DOI:** 10.1155/2016/6309484

**Published:** 2016-10-16

**Authors:** Geisa Izetti Luna, Izabel Cristina Rodrigues da Silva, Mauro Niskier Sanchez

**Affiliations:** ^1^Programa de Pós-Graduação em Saúde Coletiva, Universidade de Brasília, Brasília, DF, Brazil; ^2^FCE, Universidade de Brasília, Brasília, DF, Brazil

## Abstract

Diabetes mellitus (DM) is considered to be a worldwide epidemic disease and its type 2 form comprises more than 95% of all cases. Tumor necrosis factor-alpha (TNF-*α*) is a proinflammatory cytokine. Its dysregulation has been implicated in a variety of human diseases, including type 2 diabetes mellitus (T2DM). The control of expression of this cytokine is associated with insulin resistance and has a strong genetic influence. In order to understand this relationship, the literature from all case-control studies since 2000 to date was reviewed. The genotypes frequency results presented in ten publications with different ethnicities were compared. The correlation between the TNFA promoter genotypes and the risk of developing T2DM remains controversial due to the many discrepancies between the different studies available. Ethnic differences may play a role in these conflicting results, since the distribution of TNFA promoter polymorphisms is distinctive between individuals of dissimilar racial origin. Hence, although the relationship between T2DM incidence and presence of polymorphisms at position -308 of the TNFA gene is not entirely clear, the results of these studies suggest the need for further investigation.

## 1. Introduction

Noncommunicable diseases, including type 2 diabetes mellitus (T2DM), are considered the leading cause of death in the world and, in Brazil, constitute the main cause of diseases. T2DM assumes an important role in this context given that it is considered a worldwide epidemic disease; in 2011 it appeared among the ten leading causes of death in the world and it is prospected that number of cases will continue to increase [1]. The increase in prevalence is due to several factors. The ones most frequently being discussed are the increase in population life expectancy, changes in lifestyle (including unbalanced diet and physical inactivity), and obesity [2].

Diabetes can be defined as a group of metabolic diseases characterized by hyperglycemia resulting from defects in insulin secretion, insulin action, or both. The underlining cause of T2DM can be attributed to a combination of resistance to the action of the hormone, increased production of insulin in a compensatory manner, and inadequate secretory response [3].

On the other hand, according to a study conducted by Kaprio et al. [4], the heredity has an input around 47% in susceptibility to T2DM; thus, genetic factors play an important role in the development of the pathology [5]. Some facts corroborate the importance of heritability in T2DM: the greater concordance between monozygotic twins than among dizygotic twins and a wide variation in the prevalence of T2DM in epidemiological studies with different ethnic groups as well as positive results in numerous other genetic studies [6]. In this regard, it should be noted that even as more than 30 genes associated with T2DM have been identified, the contribution of each individual gene in the disease susceptibility is very small [7]. Additionally, most of these genes identified are related to dysfunction of pancreatic *β* cells [7].

Several reports suggest that the characteristics of each individual, including genetic polymorphisms, may confer differences in the occurrence of diabetes and other complex diseases such as cancer and heart disease [8]. The number of genes tested for the predisposition to T2DM is huge. Withal, in most cases, a positive association between polymorphisms of these genes and T2DM are not reproducible in the analysis of a different population [9]. One of these genes is TNFA that encodes the TNF-*α* protein. This molecule is composed of 157 amino acids located on chromosome 6. This cytokine is involved in inflammation, apoptosis, infections, and cancer processes [10]. Two polymorphisms in the promoter region of the TNFA gene have been described, one present at position -308 [11]. In this regard, Swaroop et al. [12] demonstrated an association between the adipocytokine TNF-*α* and development of insulin resistance.

This systematic review was conducted to provide a comprehensive assessment of the association between the polymorphism in the gene TNFA -308G/A and the presence of T2DM.

## 2. Material and Methods

### 2.1. Search Strategy

The literature search was made through the Virtual Health Library (VHL) which uses as bibliographic databases the publications found in sources such as LILACS, MEDLINE, and other information bases such as open educational resources, websites, and scientific events. Any publications with case-control studies, on January 21, 2016, that show the relationship between polymorphisms of TNFA gene -308G/A and susceptibility to type 2 diabetes mellitus (T2DM) were surveyed. The search terms used were as follows: “diabetes” and “TNF” or “tumor necrosis factor”, and “polymorphism”. The items recovered were then selected to be studied focusing on T2DM. There was no language restriction placed in the literature searched.

### 2.2. The Filters and Inclusion and Exclusion Criteria

The filters are selected in accordance with the main subjects of the following terms: “type 2 diabetes mellitus”, “tumor necrosis factor alpha”, “single nucleotide polymorphism”, and “genetic polymorphism”. The filter base on the type of study included solely the term “case-control study”. Inclusion criteria were as follows: (1) the study must have been case-control; (2) the study should have assessed type 2 diabetes mellitus and -308G/A polymorphism of TNFA gene, even if associated with other comorbidities; and (3) the study should have provided sufficient data, including the number or frequency of alleles and genotypes. Studies were excluded if (1) there were commentaries, case reports, or non-case-control or meta-analyses studies; (2) they did not report sufficient data; (3) they presented data from other types of diabetes or did not specify what kind patients had; and (4) they showed polymorphism in another coding region of the TNFA gene or (5) if the sample was composed of only DM patients.

### 2.3. Data Extraction

Data from eligible studies were extracted independently in accordance with the inclusion and exclusion criteria. For each study the following characteristics were collected: the authors, the study title, the year of publication, where it was published, the sample size, the TNFA -308G/A genotypes with their corresponding simple frequencies and percentages for the control and the case group, and the study's *p* value and odds ratio, when calculated.

## 3. Results

### 3.1. Study Characteristics

The literature search found articles published starting from 2000, given that the year the complete mapping of the human genome was made available. Advances in molecular biology technology since this project have been quick and, thus, increasing the number of genetic studies published for the association of certain genetic polymorphisms with various pathologies.

In the case of literature search for articles with case studies that related polymorphism position -308 of the TNFA gene with diabetes, the result presented forty-three publications between the years 2003 and 2015, ten of which met the criteria inclusion.

Thirteen studies presented results for other types of diabetes; eleven related to type 1 diabetes and two to gestational diabetes. Two others did not specify what type of diabetes had patients in the case group. Two of these studies did not show enough genetic data for analysis. In relation to TNFA gene, seven studies presented polymorphisms in region other than the -308 position and three publications had single nucleotide polymorphism in a gene that was not TNFA. Other studies were excluded due to different methodological designs in their inclusion criteria: two meta-analysis, one being observational and the other prospective and, relative to the sample. Furthermore, there was a publication that presented data of diabetic patient's polymorphisms in both the control and the case group.

Finally, a total of ten articles were included in this systematic review. The number and homogeneity of the included studies presented were too limited to allow a meta-analysis.

Two studies (Vendrell et al. [11], Garg et al. [18]) relate the polymorphism in gene studied with T2DM and coronary heart disease, while another two (Buraczynska et al. [14], Dabhi and Mistry [21]) relate association frequencies in diabetic patients with nephropathy or renal failure. Finally, five studies reported data that directly relate polymorphism in question with T2DM (Shiau et al. [13], Bouhaha et al. [15], Guzmán-Flores et al. [16], Saxena et al. [19], and Sefri et al. [20]), one of which also relates it with obese T2DM patients (Bouhaha et al. [15]). The general characteristics of the studies included in this review are summarized in Table 1.

### 3.2. Results of the Systematic Review

In three studies conducted in Spain, Hungary, and Morocco significant associations between the TNFA -308G/A polymorphism and the risk of T2DM were identified [11, 17, 20]: two of them in patients with other comorbidities such as coronary heart disease [11] and atherosclerosis [17].

In the present study, therefore, we infer that although there was no evidence of heterogeneity in the association between polymorphisms in the TNFA -308G/A gene and increased risk for development of T2DM, the presence of positive results may mean that few studies have not been enough to clarify this relationship, and besides there is no documented evidence in various ethnic groups. Due to contradictory results, this systematic review is aimed at providing a comprehensive assessment of the association between TNFA -308G/A and phenotype involving type 2 diabetes.

## 4. Discussion

The concern regarding the quantification and qualification of the genetic impact on phenotypic characteristics started after the first complete human sequence after genomic era [22].

As a consequence, health research delved into the genetic information as a way to find answers to complex disease questions. This type of research has shown high prevalence and increasing prospects in a number of cases, such as diabetes [23]. For this purpose, various portions of the human genome are now analyzed in search for genetic variations among individuals that could explain these diseases. Studies seeking interactions between these variations, in particular, have been very promising [24].

In the case of diabetes, a syndrome whose etiology is complicated and involves several risk factors for its development and progression, some studies on single nucleotide polymorphisms were suggested as causing the phenotypic characteristics and responsible for the increased risk of developing pathology [25–27].

To study the risk factors for a disease is to study the possibility of a certain event happening. Within the epidemiological concepts, the term is used to predict the likelihood of healthy individuals, exposed to certain factors, developing a given disease. This does not necessarily mean that it would occur; nevertheless the presence of these factors makes the individual more vulnerable to manifest the disease. A risk factor “x” may be the trigger of various diseases, and several risk factors can cooperate for the genesis of a common disease [28].

Environmental factors such as eating habits [29–31] and physical inactivity [32–34], in addition to the concomitant presence of diverse mutations in many genes, need to be considered so that the contribution of each of them is understood.

There are two types of genetic epidemiological studies: linkage studies and association. Linkage research studies genetic markers and specific polymorphisms in a family group that has the disease. This, thus, allows the researchers a way to better study the regions of a chromosome that is affected. After the connection is made to the chromosome, one can test for association of polymorphisms for identifying a genotype specific to a given disease in groups of people who have the targeted disease [34, 35].

Several association studies have discussed the importance of clarifying the relationship between genetic polymorphisms and the development of type 2 diabetes.

The search for candidate genes was initially focused on possible genes encoding proteins directly involved in the pathophysiology of T2DM: related encoders, for example, production, secretion or activity of insulin, or development of the pancreas [36–38].

Gradually, however, emerging studies suggested that several other factors could contribute to the development of T2DM, as the expression of proinflammatory cytokines and other molecules that act in the inflammatory process and that appear to play a critical role in the development of chronic complications of DM [39]. Several studies have shown the influence of various polymorphisms in proinflammatory cytokines genes as a risk of development of obesity, diabetes, and metabolic syndrome [40]. One of these cytokines, tumor necrosis factor (TNF-*α*), has been investigated since diabetics have elevated levels of it circulating. TNF-*α* has also been implicated as an insulin resistance-causing factor associated with the pathogenesis of T2DM [41].

TNF-*α* is a proinflammatory cytokine that acts in the regulation of cell proliferation, differentiation, and apoptosis [42]. Among the various polymorphisms describing the TNFA gene, the -308G>A variant located in the promoter region excels by affecting the expression of its gene [43]. The presence of the A allele at nucleotide -308 increases transcription of TNFA gene approximately twofold, therefore increasing the production of this cytokine [44]. Under this circumstance, it is expected that the polymorphic genotype may be associated with an increased frequency of diabetes and, for the proper association, of case-control studies can be used.

In this review, several studies with the frequency of genotypes in control and case group were observed for a better understanding of the population genetic profile and a possible association between polymorphism of TNFA gene and T2DM in groups with different ethnicities. The influence of genetic polymorphisms on certain diseases may be found in a population and yet not in another; this may impact the frequencies and distribution of a given polymorphism in distinct population. The difference in influence may be due to racial variantion as well as other factors. [45]. In addition, some studies have included a proportionally small sample size in relation to the country's population and most of them define the case of groups in T2DM patients who have concomitant comorbidities.

Case-control studies were selected in this review and were made with the people of Spain, Taiwan, Poland, India, Tunisia, Mexico, Hungary, and Morocco; nonetheless a positive association was only found in the Spanish, Hungarian, and Moroccan population.

Sefri et al. [20] in their meta-analysis, which considers 21 case-control studies published until August 2013, argue that no significant associations were found between the polymorphism in the studied region of the TNFA gene and risk of developing T2DM. Their finding was consistent with a previous meta-analysis, which included data from 18 association studies [46]. In these studies, publications with different ethnic groups were included; among them were Africans, Asians, Caucasians, and other populations.

So, when comparing the frequencies of polymorphic genotypes (GA and AA) in the groups of case-control study (described in Table 1), it is possible to observe that these genotypes are presented more frequently in the group that has the disease. The exceptions to this were the studies in Tunisia and India; the most frequent genotype in type 2 diabetic patients was the GG. In all other countries there was an increase of polymorphic genotype in T2DM group. This evidence supports the assumption that polymorphisms in the TNFA gene and its association with other aspects, both genetic and environmental, may represent an important risk factor for type 2 diabetes mellitus.

## 5. Conclusion

Risk factors for T2DM are increasingly more prevalent in the population. Risk factors not modifiable or irreversible in nature, such as genetic profiles, refer to the individual characteristics. Even though these factors may not be changed, the identification of their presence in individuals and families may enable health professionals to advise change in the lifestyle of these patients, avoiding early manifestation of T2DM.

## Figures and Tables

**Table 1:** Comparison of type 2 diabetes mellitus case-control study results for the -308 TNFA gene.

Author	Study	Year	Country/ Ethnicity	Sample	TNFA -308 polymorphism	Case group frequency		Control group frequency		*p* value	Odds ratio (in article)
Vendrell et al. [11]	A polymorphism in the promoter of the tumor necrosis factor-*α* gene (/308) is associated with coronary heart disease in type 2 diabetic patients	2002	Spain	*n* = 313	GG GA + AA	63 (59.4%) 43 (40.6%)		159 (76.8%) 48 (23.2%)		*p* = 0.0056^*∗*^	OR = 1.96

Shiau et al. [13]	TNF-*α* polymorphisms and type 2 diabetes mellitus in Taiwanese patients	2003	Taiwan	*n* = 444	GG GA AA	218 (84.82%) 35 (13.62%) 4 (1.56%)		168 (89.84%) 16 (8.56%) 3 (1.60%)		*p* = 0.2343	—

Buraczynska et al. [14]	Genetic determination of TNF and myeloperoxidase production in dialyzed patients with diabetic nephropathy	2004	Poland	*n* = 210				Healthy	Other nephropathy	*p* = 0.07 (case versus control – healthy)	—
					TNF1/TNF1 (GG)	22 (59.5%)		86 (74.8%)	40 (69%)		
					TNF1/TNF2 (GA)	13 (35.1%)		24 (20.9%)	15 (25.8%)		
					TNF2/TNF2 (AA)	2 (5.4%)		5 (4.3%)	3 (5.2%)		

Bouhaha et al. [15]	Study of TNF-*α* -308G/A and IL6 -174G/C polymorphisms in type 2 diabetes and obesity risk in the Tunisian population	2010	Tunisia	*n* = 494	GG GA AA	141 (72.3%) 51 (26.1%) 3 (1.6%)		204 (68.2%) 89 (29.7%) 6 (2.1%)		*p* = 0.34	—

Guzmán-Flores et al. [16]	Tumor necrosis factor-alpha gene promoter -308G/A and -238G/A polymorphisms in Mexican patients with type 2 diabetes mellitus	2011	Mexico	*n* = 904	GG GA AA	225 (86.9%) 31 (12.0%) 3 (1.1%)		573 (88.8%) 69 (10.7%) 3 (0.5%)		*p* = 0.558 (GG versus GA) *p* = 0.238 (GG versus AA)	OR = 1.14 (GG versus GA) OR = 2.55 (GG versus AA)

Szabó and Acsády [17]	Tumor necrosis factor-*α* 308 GA polymorphism in Atherosclerotic patients	2011	Hungary	*n* = 314		DM + AVE	DM + MI			*p* < 0.05^*∗*^ (control versus DM + MI) *p* < 0.005^*∗*^ (control versus DM + AVE)	OR = 6.71 (DM + MI) OR = 5.87 (DM + AVE)
					GG	40 (61.6%)	42 (64.6%)	141 (76.7%)			
					GA	20 (30.7%)	17 (26.2%)	40 (21.7%)			
					AA	5 (7.7%)	6 (9.2%)	3 (1.6%)			

Garg et al. [18]	Proinflammatory cytokine gene polymorphisms and threat for coronary heart disease in a North Indian Agrawal population	2012	Indian	*n* = 322	GG GA AA	117 (85.4%) 20 (14.59%) 0		146 (78.9%) 38 (20.54%) 1 (0.54%)		*p* = 0.27	OR = 1.08 (GA/AA versus GG)

Saxena et al. [19]	Association of IL-6, TNF-*α*, and IL-10 gene polymorphisms with type 2 diabetes mellitus	2013	Indian	*n* = 353	GG GA AA	173 (81.2%) 33 (15.5%) 7 (3.3%)		111 (79.3%) 25 (17.9%) 4 (2.9%)		*p* = 0.3766	OR = 1.07 (allele G versus A)

Sefri et al. [20]	TNFA -308G>A polymorphism in Moroccan patients with type 2 diabetes mellitus: a case-control study and meta-analysis	2014	Morocco	*n* = 551	GG GA AA	38 (12.38%) 154 (50.16%) 115 (37.56%)		60 (24.59%) 133 (54.51%) 51 (20.90%)		*p* = 0.000002^*∗*^ (allele G versus A)	OR = 1.79 (allele G versus A)

Dabhi and Mistry [21]	Oxidative stress and its association with TNF-*α* -308G/C and IL-1*α* -889C/T gene polymorphisms in patients with diabetes and diabetic nephropathy	2015	Indian	*n* = 449	GG GA AA	185 (86.40%) 27 (12.62%) 2 (0.97%)		191 (81.11%) 44 (18.88%) 0		*p* = 0.41	OR = 0.75

^*∗*^ *p* < 0,005.
